# Biomarker Value in the Diagnosis of Community-Acquired Pneumonia with Concomitant Chronic Heart Failure

**DOI:** 10.3390/jcm10194570

**Published:** 2021-10-01

**Authors:** Svetlana Rachina, Andrey Bobylev, Pavel Lazarev, Vladimir Mladov, Florence Carrouel, Sergey Avdeev, Roman Kozlov, Denis Bourgeois

**Affiliations:** 1 Internal Medicine Department, Sechenov First Moscow State Medical University, 119991 Moscow, Russia; 2 Institute of Antimicrobial Chemotherapy, State Medical University, 214019 Smolensk, Russia; andrey.bobylev@antibiotic.ru (A.B.); roman.kozlov@antibiotic.ru (R.K.); 3 Pharmaceuticals Division, Bayer JSC, 117198 Moscow, Russia; spaceman665@gmail.com; 4 Market Access Department, CSC BIOCAD, 198515 Saint-Petersburg, Russia; vmladov@ya.ru; 5 Health, Systemic, Process, EA 4129 Research Unit, University Claude Bernard Lyon 1, University of Lyon, 69008 Lyon, France; florence.carrouel@univ-lyon1.fr (F.C.); denis.bourgeois@univ-lyon1.fr (D.B.); 6 Pulmonology Department, Sechenov First Moscow State Medical University, 119991 Moscow, Russia; serg_avdeev@list.ru

**Keywords:** community-acquired pneumonia, chronic heart failure, inflammatory biomarkers

## Abstract

The diagnosis of community-acquired pneumonia (CAP) with chronic heart failure (CHF) is associated with objective difficulties. Our case–control study aims to establish whether established serum inflammatory biomarkers are relevant to the diagnosis of CAP in patients with CHF. Seventy inpatients with previously diagnosed CHF and suspected non-severe CAP were recruited and then stratified into two subgroups with confirmed and rejected diagnosis of CAP. C-reactive protein (CRP), procalcitonin (PCT), tumor necrosis factor α (TNFα), interleukin-6 (IL-6) and brain natriuretic peptide (BNP) were measured. The value of biomarkers was determined using logistic regression, and their discriminatory efficacy was assessed by analyzing receiver operating characteristic (ROC) curves. Significantly higher levels of CRP 50.0 (35.5–98.5) mg/L, PCT 0.10 (0.05–0.54) ng/mL and IL-6 46.1(21.4–150.3) pg/mL in cases were identified as compared to the control group—15.0 (9.5–25.0) mg/L, 0.05 (0.05–0.05) ng/mL and 13.6 (9.5; 25.0) pg/mL, respectively. The Area Under the ROC Curve (95% CI) was the highest for CRP—0.91 (0.83–0.98), followed by PCT—0.81 (0.72–0.90) and IL-6—0.81 (0.71–0.91). A CRP value of >28.5 mg/L had an optimal sensitivity and specificity ratio (85.7/91.4%). In conclusion, the measurement of serum CRP, PCT and IL-6 levels can be useful for the diagnosis of CAP in patients with CHF. CRP showed optimal diagnostic utility in this population.

## 1. Introduction

Community-acquired pneumonia (CAP) is one of the most common acute infections requiring admission to hospital. The main causative pathogens of CAP are *Streptococcus pneumoniae* (*S. pneumoniae*), *Influenza A*, *Mycoplasma pneumoniae* and *Chlamydophila pneumoniae*, and the main risk factors are age, smoking and comorbidities. CAP disproportionately affects persons who are very young or very old, with an annual incidence of 9.2 to 33 per 1000 person-years [1,2]. Out of an estimated 878,000 adults 45 years and older who were hospitalized with a primary diagnosis of CAP in 2010, 71% were 65 years or older, and 10% to 20% required admission to the intensive care unit (ICU) [3]. 

Although the diagnosis and treatment of CAP are straightforward in most cases, they can be more complex, and recent data indicate that the mortality of CAP in the UK is surprisingly high. Although *S. pneumoniae* remains the most commonly isolated pathogen in CAP, the relative frequency of other pathogens has increased. Clinical suspicion should be driven by comorbidities and other risk factors [4]. Commonly used diagnostic methods may identify a pathogen in only 30% to 40% of patients [5]. The diagnosis of CAP in chronic heart failure (CHF) patients is also fraught with objective difficulties as CHF can mask the clinical signs of CAP [6,7]. Importantly, typical signs of CHF can also be explained by a new episode of respiratory tract infection (RTI) including CAP [8].

Symptoms and signs lack sensitivity and specificity, and chest radiography is not very accurate [9]. The sensitivity of chest X-ray (CXR) is poor and interobserver agreement for the presence of pneumonia is limited, with κ coefficients ranging from 0.37 to 0.53 [10]. This translates into a low confidence of clinicians toward CXR results [9], leading to a poor predictive value of clinical data for the diagnosis of CXR or computed tomography (CT)-proven pneumonia [11]. Therefore, an initial diagnosis of pneumonia has limited predictive value for the presence of pneumonia.

The overdiagnosis of pneumonia leads to the inappropriate use of antibiotics and may delay appropriate management of mimetic disease. Alternative imaging strategies, including CT or pulmonary ultrasound, may improve the diagnosis of pneumonia. CXR is distinguished by relatively low specificity for CAP verification in the population of patients with CHF [12].

Patients with CAP should be assessed for disease severity using the CURB-65 score (or its derivative CRB65) [13]. The CURB-65 score dictates optimum empirical antibiotic therapy, and whether the patient can be treated safely at home (scores of 0 or 1) or should be admitted to a general (a score of 2) or possibly an intensive care ward (a score of 3+). Although it has been extensively validated and is simple to use, the CURB-65 score has limitations. It is poor at detecting the need for intensive care, with only 51% of patients requiring admission to intensive care units having CURB-65-defined severe disease (a score of 3+) [14]. In addition, 20% of deaths occur in patients with CURB-65 scores of 2 or lower, and there was a mortality of 8% in the BTS audit for patients with a score of 2.4.

Biomarkers may facilitate clinical decisions in order to guide antimicrobial treatment and the prediction of prognosis in community-acquired pneumonia. For optimal management and use of healthcare resources in CAP, early identification of the causative agent(s), recognition of disease severity and prediction of unfavorable outcome are of major importance [15]. Biomarkers, applied in synergy with clinical assessment and CAP-specific severity scores, may provide additional information on disease severity and on the distinction between bacterial and viral etiology.

Inflammatory biomarkers are undoubtedly among the crucial molecules in the pathogenesis of CAP, where the most studied are C-reactive protein (CRP), procalcitonin (PCT), tumor necrosis factor α (TNFα) and interleukins, namely IL-6. The utilized markers of cardiac dysfunction include creatine kinase-muscle/brain, troponins, copeptin, pro-adrenomedullin, atrial natriuretic peptide and B-type natriuretic peptide (BNP). Parallel measurement of biomarkers of different profiles may prove useful in the cases of coexistent CHF and CAP [16,17,18].

Our research aims to establish whether established serum inflammatory biomarkers are relevant to the diagnosis of CAP in patients with CHF. One specific question is addressed as follows according to the PICO principles: In adults with CHF, does measuring established serum inflammatory biomarkers improve the diagnosis of CAP?

## 2. Materials and Methods

### 2.1. Study Design

This study was designed as a case–control study conducted at the two primary care hospitals in Smolensk, Russia. This research was performed in accordance with the STROBE guidelines (Supplementary Materials Table S1). 

**Figure 1:**
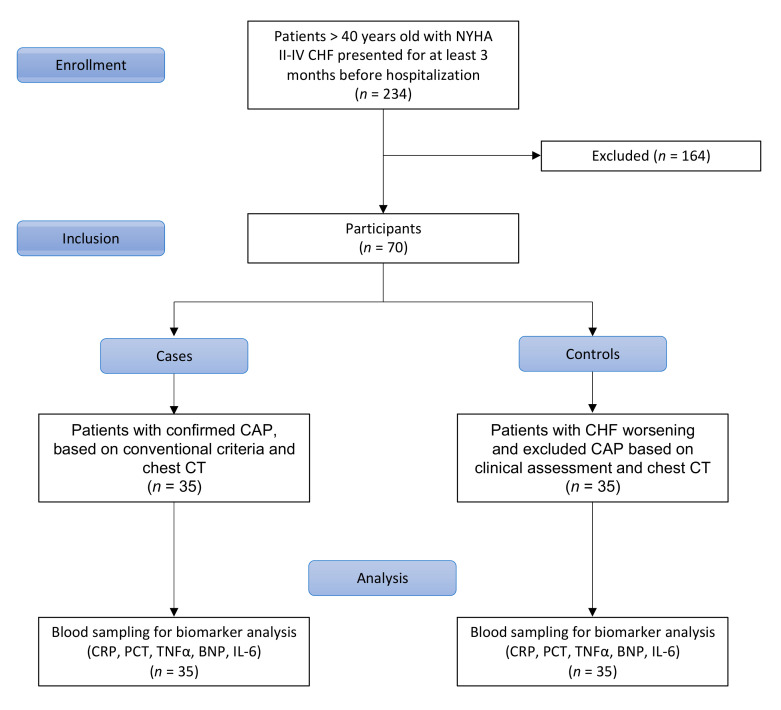
Flowchart of the study.

The study comprised 70 patients, aged over 40 years, from a hospitalized pool, between September 2013 and September 2014. The inclusion process is presented in Figure 1. Consecutive class II–IV CHF individuals for at least 3 months before hospitalization and supported by previous medical documentation were included. CAP diagnosis was based on complex clinical assessment by the experienced clinician in line with national guidelines [19]. Thirty-five non-severe CAP patients defined by (1) a new pulmonary infiltrate on chest CT, (2) acute fever >38.0 °C) and (3) at least one of the following symptoms or signs: cough (productive or nonproductive), dyspnea, respiratory chest pain, crackles or reduced respiratory sounds, were recruited during the period. Meanwhile, 35 persons without CAP but having signs and symptoms of worsening CHF were assigned to the control group for a comparative study (Ratio 1:1). The control group was matched to cases on age (within 5 years). Patients with CAP were considered cases, persons without CAP but having signs and symptoms of CHF decompensation were seen as controls.

### 2.2. Classification

Guidelines from the Infectious Diseases Society of America advise diagnosing CAP based on suggestive examination findings and characteristic infiltrate on chest radiography with or without microbiologic data [20]. 

Patients’ heart failure classifications are determined by a doctor based on their heart failure symptoms and functional limitations. The measure of the severity is based on the New York Heart Association Class guidelines. New York Heart Association functional class II–IV CHF includes:

Class II (Mild): Patients with cardiac disease resulting in slight limitation of physical activity. They are comfortable at rest. Ordinary physical activity results in fatigue, palpitation, dyspnea or anginal pain.

Class III (Moderate): Patients with cardiac disease resulting in marked limitation of physical activity. They are comfortable at rest. Less than ordinary activity causes fatigue, palpitation, dyspnea or anginal pain.

Class IV (Severe): Patients with cardiac disease resulting in the inability to carry on any physical activity without discomfort. Symptoms of heart failure or the anginal syndrome may be present even at rest. If any physical activity is undertaken, discomfort is increased.

Chest CT was obtained upon admission and interpreted in a blinded fashion by 3 observers and classified as definite, normal or uncertain for CAP. Bayesian analysis was applied to assess the accuracy of chest radiograph in the diagnosis of pneumonia.

### 2.3. Blood Sampling

Blood samples were obtained at hospital admission with serum and plasma samples drawn into pyrogen-free vacutainer tubes. Tubes for plasma samples contained EDTA as an anticoagulant. Serum or plasma was separated from whole blood within 60 min by refrigerated centrifugation at 2000× *g* for 15 min and stored in several aliquots at −70 °C.

### 2.4. Biomarker Analysis

The CRP was measured using photometric turbidimetry immunoassay with semiautomatic analyzer Humalyzer 2000 (HUMAN, GmbH, Germany) and HUMAN diagnostic kits. PCT was measured using chemiluminescence method with automatic mini-VIDAS analyzer (BioMerieux, Marcy-l’Étoile, France) and BioMerieux diagnostic kits (VIDAS BRAHMS). Cytokines (IL-6 and TNFα) were measured with the use of solid-phase ‘sandwich’ variant of enzyme linked immunosorbent assay (ELISA) by means of diagnostic kits Interleukin-6-IFA-Best and alfa-FNO-IFA-BEST (CJSC «Vector Best», Novosibirsk, Russia) and Multiscan EX IFA-reader («Thermo Labsystems», Beijing, China). BNP measurement was made using chemiluminescence method with automatic analyzer ADVIA Centaur XP (Siemens, New York, NY, USA), Siemens diagnostic kits (closed system) and assayed BIO-RAD controls (USA).

### 2.5. Biomarker Reference

Reference values of biomarkers were as follows: CRP ˂ 8 mg/L or equal, PCT less than 0.1 ng/mL, IL-6 < 10 pg/mL or equal, TNFα < 6 pg/mL or equal, BNP 0–80 pg/mL for patients under 60 and 0–100 pg/mL for those >60 years.

### 2.6. Statistical Analysis

Statistical data processing was performed with the use of R v.3.3.2 free software environment for statistical computing and graphics (The R Foundation for Statistical Computing, Vienna, Austria). Based on the sample size, the distribution of numerical variables was considered to be abnormal. Summary statistical data are presented as the number of observations, median and interquartile range (IQR) with 25th–75th percentile values. Intra-group comparisons were carried out by means of Wilcoxon test for paired samples with Holm–Bonferroni adjustment for multiple comparisons. Mann–Whitney test was applied to study inter-group differences of continuous variables. Categorical variables are presented as absolute and relative rates that were compared using Fisher’s exact test. Sensitivity and specificity for each biomarker are presented. CRP cut-off >28.5 mg/L was chosen to achieve the optimal balance between sensitivity and specificity, while giving priority to sensitivity, since the risk of underdiagnosis of CAP and inadequate treatment in terms of clinical consequences is more serious than overdiagnosis, although this can lead to over-prescription of antibiotics. Deviations into higher or lower CRP value will significantly degrade either sensitivity or specificity. The statistical significance of biomarkers in the diagnosis of CAP was determined using logistic regression. First, the independent efficacy of each parameter was measured by constructing univariable predictor models. Discriminatory efficacy was assessed by analyzing receiver operating characteristic (ROC) curves and was numerically interpreted using Area Under the ROC Curve (AUC) values. All differences were considered to be statistically significant when the *p*-value was less than the level of significance (0.05) in two-tailed tests.

## 3. Results

### 3.1. Baseline Characteristics

Our study included 35 cases and 35 controls—median (IQR) age 78 (64–82) and 77 (71–82) years old, respectively. Baseline demographic and clinical characteristics of patients are presented in Table 1. All of those parameters showed no significant differences between the study groups except for body temperature, which was mildly elevated in the group of cases.

### 3.2. Biomarker Levels at Study Time Point

**Figure 2:**
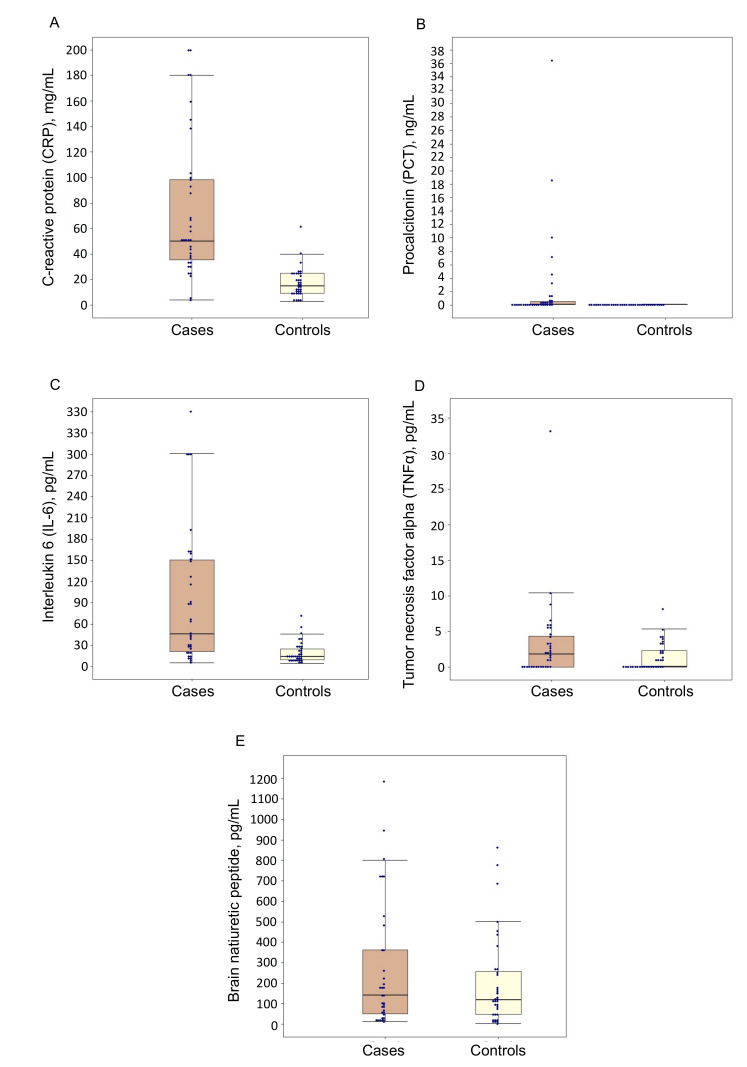
Serum C-reactive protein (**A**), procalcitonin (**B**), interleukin 6 (**C**), tumor necrosis factor α (**D**) and brain natriuretic peptide (**E**) concentrations in patients with (cases) or without (controls) community-acquired pneumonia. Each box represents the first quartile, median quartile and third quartile, from bottom to top.

The results of the measurements of serum CRP, PCT, IL-6, TNFα and BNP concentrations are presented in Figure 2. The value of CRP was 50.0 (35.5–98.5) vs. 15.0 (9.5–25.0) mg/L in cases and controls, respectively; the between-group comparisons showed significant differences. Serum PCT and IL-6 levels were also markedly elevated in patients with CAP during observation—0.10 (0.05–0.54) ng/mL and 46.1 (21.4–150.3) pg/mL, respectively—and significantly different from those in the control group (*p* < 0.0001 for both comparisons).

For TNFα, there were no statistically significant differences in the concentrations of this biomarker during between-group comparisons with all median values in both groups being within the reference range.

The concentration of BNP was 141.2 (51.6–362.1) pg/mL in cases vs. 117.6 (47.8–256.8) pg/mL in the control group. There were no differences in the values of this biomarker between the two groups.

**Figure 3:**
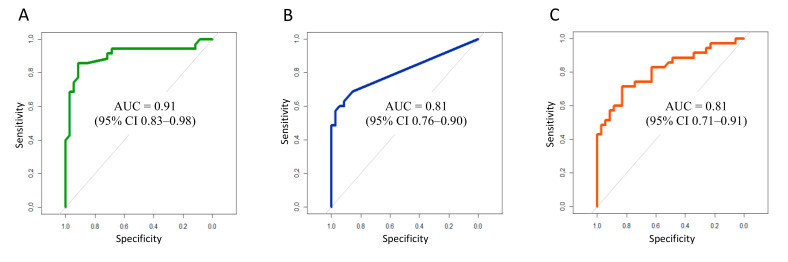
Receiver operating characteristic curve showing the discriminatory value of CRP (**A**), PCT (**B**), IL-6 (**C**) for the prediction of community-acquired pneumonia in chronic heart failure patients.

After performing regression analyses using five individual models for each predictor, CRP (*p* = 0.0001), PCT (*p* = 0.042) and IL-6 (*p* = 0.003) demonstrated significant associations with the presence of CAP, while TNFα (*p* = 0.092) and BNP (*p* = 0.339) did not bear any relationship to this diagnosis. Figure 3A–C display ROC curve analysis results, showing discriminatory abilities for the promising biomarkers (CRP, PCT and IL-6). Quantitatively, we obtained the following AUC measures: 0.91 (95% CI 0.83–0.98) for CRP, 0.81 (95% CI 0.72–0.90) for PCT and 0.81 (0.71–0.91) for IL-6. Thus, the diagnostic accuracy of CRP can be considered excellent (AUC ≥ 0.90), while PCT and IL-6 demonstrated good discrimination (AUC ≥ 0.80). 

We measured the optimal cut-off values for the aforementioned biomarkers, allowing us to confirm/exclude CAP with adequate sensitivity and specificity in the population of patients with coexisting CHF. Selected concentrations of each predictor with respective efficacy measures are presented in Table 2.

## 4. Discussion

The purpose of our work was to investigate various serum biomarkers in the diagnosis of CAP in a cohort of patients with concomitant CHF, where diagnosis by standard criteria, including chest X-ray, is challenging. Recent systemic review showed the lack of consensus for recommended reliable markers in distinguishing lung congestion and lung injury, including those associated with infection, confirming that this is still an unmet need [21]. Our study managed to propose such a simple and inexpensive biomarker as CRP, which is routinely available in clinical practice. Its value in reducing the excessive antibiotic use in CHF patients with suspected non-severe CAP was further proven in a randomized clinical trial [22].

The challenges of CAP diagnosis in the presence of concomitant CHF can be attributed to the difficulties of the assessment of symptoms common to both cardiovascular and respiratory diseases. Routine laboratory diagnosis of CAP in CHF patients is fraught with some difficulties, since systemic inflammatory response can be variable [16,23,24]. Conventional CXR is still considered the standard diagnostic method for CAP. However, the rate of misdiagnosis in adults with the suspicion of CAP reaches 30% and increases with age [12]. 

According to the results of our study, the most effective predictor of CAP in patients with concomitant CHF was CRP. The diagnostic value of PCT and IL-6 was considered to be inadequate as both these biomarkers are characterized by marked inter-individual variability of the measurements. In our study, the CRP value of >28.5 mg/L was an optimal cut-off to verify CAP in patients with concomitant CHF with 85.7% sensitivity and 91.4% specificity. A number of authors suggested different cut-off values for the diagnosis of CAP in uni- and multivariable models with varying measures of overall efficacy [25,26,27,28]. A cut-off value of 50 mg/L is most widely reported, although its sensitivity/specificity ratio varied between different studies. Some authors also suggested higher CRP cut-off values (100–200 mg/L), allowing them to confirm or exclude the presence of pneumonia with various sensitivity and specificity [25,28]. 

CRP is the most studied marker of bacterial inflammation and one of the most validated diagnostic laboratory tests in clinical practice worldwide. In our study, 97% of patients with CAP had at least a twofold elevation of the serum CRP level above reference values. The median serum CRP concentration was relatively low compared to the data obtained by other authors [25]. These findings may be attributed to the age-related dysregulation of pro-inflammatory mediators and the consequent disruption of protein synthesis in hepatocytes in seniors, who prevailed in our study. It is worth mentioning that we recruited patients with suspected non-severe CAP. 

Despite the fact that the etiology of CAP was not a subject of the study, pathogens were identified in 15/35 (42.9%) cases. *S. pneumoniae* was the most common pathogen (10/15 cases, 8/10-monoinfection, 2/10 co-infection with respiratory viruses), and there were isolated cases of other bacterial pathogens, such as *Haemophilus influenzae*, Enterobacterales and *Staphylococcus aureus*.

The hypersecretion of CRP is registered during other conditions including CHF [29]. Serum CRP in patients with excluded CAP was also elevated in our study, with median values as high as 15.0 mg/L. The between-group differences were statistically significant, pointing to the possible use of this biomarker as a means of CAP verification in the presence of concomitant CHF. Other authors obtained similar results [16,24]. Thus, in the study by Lee Y.J. et al. in patients with cardiac congestion, the CRP value was 12 (0.4–247.4) mg/L, while in patients with respiratory disease its values amounted to 103 (0.2–464.9) mg/L [23]. According to Cha Y.S. et al., the median CRP concentrations in patients with pneumonia greatly exceed those in patients with isolated CHF (61.0 mg/dL vs. 5.6 mg/L, *p* < 0.001) [6]. 

Regression analysis confirmed the diagnostic value of CRP for the prediction of CAP in CHF patients with excellent discriminatory ability (AUC = 0.91; 95% CI 0.83–0.98). Our results match those previously demonstrated by other authors [16,23,25,26].

Alongside CRP, PCT is a more specific biomarker of acute generalized bacterial inflammation [30]. The overproduction of PCT, associated with the development of pneumonia, has been previously described; however, the exact values differed significantly [31,32]. The baseline level of PCT, identified in our study, turned out to be highly variable and exceeded 2 ng/mL in only 17% of the study participants. 

PCT readings in CHF patients are also variable. Our results concur with the latter findings: PCT measurements in CHF patients without CAP were lower than 0.1 ng/mL in almost all cases. 

Such contradictory findings can be attributed to the differences in baseline characteristics of CHF patients. Significant PCT elevation may be due to the predominance of patients with marked left ventricular (LV) systolic dysfunction in those samples [33]. In our study, conversely, participants had preserved or mildly decreased LV function.

Regression analysis demonstrated high PCT efficacy for the diagnosis of CAP in CHF patients with AUC = 0.81 (95% CI 0.72–0.90). According to the literature, the predicting value of PCT for the diagnosis of CAP in different patient populations is, nevertheless, relatively variable [7,18,26,27]. In one study, the measurement of PCT in addition to the clinical model did not prove to be useful in patients with acute cough [27]. The significance of PCT as a predictor of low RTI/CAP in CHF patients varied from AUC = 0.72 to AUC ˃ 0.8, being, in general, comparable to the results of our study [7,18].

IL-6 is a typical proinflammatory cytokine, which is elevated in various infectious and non-infectious diseases. Its mean concentration increases in CAP cases in the range of 48 pg/mL to 3569 pg/mL, being highly variable due to different study designs, although not usually exceeding 300 pg/mL [17,34]. The role of cytokines, including IL-6, in the realization of molecular mechanisms of cardiac dysfunction has been proven [35]. However, in the majority of published studies, this parameter amounted only to 10–15 pg/mL, slightly out of the reference range [36,37]. The baseline IL-6 level in our study exceeded reference values, but matched the previously mentioned range. In comparison, the IL-6 level was significantly higher in the group of patients with CAP than in those CHF patients without CAP. A similar tendency was found in the studies by Mueller T. et al. and Wang W. et al. [17,18]. The diagnostic value of IL-6 for CAP verification in concomitant CHF was shown using logistic regression with good discriminatory accuracy (AUC = 0.81; 95% CI 0.72–0.90).

TNFα is a multifunctional proinflammatory cytokine. In CAP patients, the published results ranged from being close to normal to greatly elevated [38,39]. In our study, TNFα concentrations in confirmed CAP demonstrated marked variability, but remained within the reference range. The predominance of non-severe CAP could explain these results. 

The role of TNFα in the pathogenesis of CHF has been demonstrated before, but the degree of its effect on the development and progression of the disease is not yet completely determined. Normal and elevated mean values of biomarkers in CHF were detected in relevant studies with similar frequency [38,40]. In our study, TNFα levels were comparable in both groups. Regression analysis confirmed the inefficacy of this biomarker for CAP verification in comorbid CHF.

BNP is considered to be the ‘gold-standard’ biomarker of CHF development and progression, although its concentration could rise in other illnesses as well, while still being associated with cardiovascular injury in most cases. The variability of published results in CAP (mean BNP level from 26 ± 15 pg/mL to 273 ± 36 pg/mL) could be attributed to the presence of concomitant CHF in a certain proportion of patients, as they were not formally excluded. Komiya et al. evaluated the usefulness of BNP and CRP for distinguishing an acute lung injury/acute respiratory distress syndrome (ALI/ARDS) from cardiogenic pulmonary edema (CPE) in a cross-sectional study [41]. Although both CRP and BNP had good discriminatory performance, the combination showed advantages over each of them. It should be noted that BNP was significantly elevated in mixed ALI/ARDS and CPE cases. This correlates to a certain extent with our findings.

Due to the inclusion of patients with CAP and confirmed CHF in the study, serum BNP concentrations were logically elevated and amounted to 141.2 (51.6–362.1) pg/mL. Similar results were obtained in the group of CHF patients without CAP. The literature data demonstrate the wide variation of this parameter in patients with CHF [42,43]. This phenomenon is attributed to the inter-individual variability of BNP levels in different CHF populations, determined by the type, stage and the degree of CHF compensation, extracardiac factors and other diseases [37,44,45]. 

The BNP values obtained for CHF patients in our study, irrespective of the presence of CAP, did not deviate from the previously mentioned range of values. The lack of difference in BNP readings points to the futility of its additional use for CAP diagnostics in this patient category. 

A limitation of our study was the relatively small sample size, which allows us to regard the obtained results as preliminary, requiring verification in larger studies. The study would also be strengthened by using a validation cohort, although our algorithm based on proposed CRP cut-off >28.5 mg/L was validated further in a randomized prospective controlled study that recruited 76 patients in each arm [22]. It proved the hypothesis that additional measurement of serum CRP in patients with CHF and suspected non-severe CAP with a cut-off >28.5 mg/L reduces the rate of systemic antibacterial therapy without the worsening of outcomes. The strengths of our study include the careful selection of patients and verification of CAP by chest CT, which is characterized by a high value in distinguishing infiltrates and pulmonary congestion. Furthermore, the possible use of such a simple and affordable biomarker as CRP will contribute to reducing excessive antibiotic use and the burden of antibiotic resistance. 

## 5. Conclusions

Clinical features of CAP in CHF hamper the diagnostic process, providing the impetus for the search for affordable, effective and safe confirmatory diagnostic methods. Such serum biomarkers as CRP, PCT and IL-6 were significantly elevated in CAP in comparison to isolated CHF. The measurement of TNFα and BNP in this scenario had no practical value. CRP was proven to be the most useful diagnostic biomarker with an elevation of >28.5 mg/L, allowing us to verify the presence of CAP in patients with concomitant CHF with sufficiently high sensitivity (85.7%) and specificity (91.4%).

## Figures and Tables

**Table 1:** Baseline characteristics of patients.

	Cases (*n* = 35)	Controls (*n* = 35)	*p*
Female sex, *n* (%)	24 (68.6)	22 (62.9)	>0.05
Median age, years	78 (64–82)	77 (71–82)	>0.05
Time since CHF diagnosis, months	65 (48–138)	64 (49–125)	>0.05
Number of CHF worsening episodes during past year	2 (1–2)	2 (1–2)	>0.05
CHF functional class, *n* (%)			
II III IV	21 (60.0)	17 (48.6)	
	12 (34.3)	17 (48.6)	>0.05
	2 (5.7)	1 (2.8)	
Cough, *n* (%)	33 (94.3)	31 (88.6)	>0.05
Sputum production, *n* (%)	14 (40)	12 (34.3)	>0.05
Dyspnea, *n* (%)	35 (100)	35 (100)	>0.05
Body temperature, °C	36.8 (36.6–37.2)	36.4 (36.3–36.8)	0.0005
Edema, *n* (%)	29 (82.9)	28 (80)	>0.05
Edema location, *n* (%) Feet only Feet and shins Anasarca			
	4 (13.8)	8 (28.6)	>0.05
	25 (86.2)	19 (67.9)	
	0 (0)	1 (3.5)	
Fine crackles/rales on auscultation, *n* (%)	33 (94.3)	30 (85.7)	>0.05
Dry rales on auscultation, *n* (%)	7 (20)	5 (14.3)	>0.05
Infiltration on chest X-ray, *n* (%)	28 (80)	32 (91)	>0.05
Pleural effusion on chest X-ray, *n* (%)	23 (65.7)	15 (42.9)	>0.05
Peripheral WBC count (×10^9^ L)	8.4 (7.0–11.2)	8.3 (7.0–9.5)	>0.05
Band neutrophils, *n* (%)	7 (5.0–9.8)	6 (1.0–10.0)	>0.05
In-hospital mortality, %	2.9	2.9	>0.05

**Table 2:** Sensitivities and specificities of selected cut-off values of most promising biomarkers for diagnosis of CAP in CHF patients. Sn: Sensitivity; Sp: Specificity.

CRP			PCT			IL-6		
Sn (%)	Sp (%)	Cut-off (mg/L)	Sn (%)	Sp (%)	Cut-off (ng/mL)	Sn (%)	Sp (%)	Cut-off (pg/mL)
100.0	8.6	3.5	68.6	85.7	0.050	88.6	48.6	13.3
97.1	11.4	5.0	62.9	91.4	0.055	85.6	48.6	13.5
94.3	11.4	7.0	60.0	91.4	0.065	85.7	51.4	13.9
94.3	25.7	9.5	60.0	94.3	0.075	82.9	54.3	14.4
94.3	34.3	11.5	57.1	97.1	0.085	82.9	57.1	15.1
94.3	48.6	14.5	54.3	97.1	0.095	82.9	60.0	15.9
94.3	57.1	17.5	48.6	97.1	0.115	80.0	62.9	18.7
94.3	68.6	21.0	48.6	100.0	0.145	74.3	65.7	20.2
91.4	71.4	23.5	45.7	100.0	0.185	74.3	68.6	21.5
88.6	71.4	24.5	42.9	100.0	0.220	74.3	74.3	23.9
85.7	85.7	25.5	40.0	100.0	0.235	71.4	77.1	26.3
85.7	88.6	26.5	37.1	100.0	0.265	71.4	82.9	27.6
85.7	91.4	28.5	34.3	100.0	0.335	65.7	82.9	28.4
80.0	91.4	31.5	31.4	100.0	0.395	60.0	82.9	31.9
77.1	91.4	33.5	28.6	100.0	0.460	60.0	85.7	35.7
74.3	94.3	35.5	25.7	100.0	0.545	57.1	88.6	39.3
71.4	94.3	38.0	22.6	100.0	0.945	54.3	91.4	42.5
68.6	97.1	40.5	20.0	100.0	1.355	51.4	91.4	44.3
62.9	97.1	45.0	17.1	100.0	2.255	48.6	94.3	49.9
60.0	97.1	48.0	14.3	100.0	3.845	48.6	97.1	58.5
45.7	97.1	54.0	11.4	100.0	5.855	42.9	98.1	67.7
42.9	97.1	60.0	8.6	100.0	8.665	42.9	100.0	78.8
40.0	100.0	64.0	5.7	100.0	14.44	40.0	100.0	87.3
37.1	100.0	67.0	2.9	100.0	27.56	37.1	100.0	88.9

## Data Availability

The data presented in this study are available on request from the corresponding author.

## Supplementary Materials

The following are available online at https://www.mdpi.com/article/10.3390/jcm10194570/s1, Table S1: STROBE Statement—checklist of items.
